# *Phytophthora* Species Involved in *Alnus glutinosa* Decline in Portugal

**DOI:** 10.3390/pathogens12020276

**Published:** 2023-02-08

**Authors:** Carlo Bregant, Eduardo Batista, Sandra Hilário, Benedetto T. Linaldeddu, Artur Alves

**Affiliations:** 1 Dipartimento Territorio e Sistemi Agro-Forestali, Università degli Studi di Padova, Viale dell’Università, 16, 35020 Legnaro, Italy; 2 CESAM, Departamento de Biologia, Universidade de Aveiro, 3810-193 Aveiro, Portugal

**Keywords:** emerging diseases, invasive pathogens, pathogenicity

## Abstract

Recent field surveys conducted in five common alder ecosystems in Portugal have shown the occurrence of severe canopy dieback, bleeding canker and root rot symptoms indicative of *Phytophthora* infections. Isolations from symptomatic tissues, rhizosphere and water samples yielded a total of 13 *Phytophthora* species belonging to 6 phylogenetic clades, including *P. lacustris* (13 isolates), *P. multivora* (10), *P. amnicola* (9), *P. chlamydospora* (6), *P. polonica* (6), *P. bilorbang* (4), *P. plurivora* (4), *P. cinnamomi* (3), *P. asparagi* (2), *P. cactorum* (2), *P. pseudocryptogea* (2), *P. gonapodyides* (1) and *P. rosacearum* (1). Results of the pathogenicity test confirmed the complex aetiology of common alder decline and the additional risk posed by *Phytophthora multivora* to the riparian habitats in Portugal. At the same time, the diversity of *Phytophthora* assemblages detected among the investigated sites suggests that different species could contribute to causing the same symptoms on this host. Two species, *P. amnicola* and *P. rosacearum,* are reported here for the first time in natural ecosystems in Europe.

## 1. Introduction

Alders represent an important component of European riparian and wetland vegetation. In Europe, four alder species grow spontaneously, mainly along rivers, streams and damp environments, often with a pioneer behaviour fundamental for ecological succession [1,2]. In Portugal, the only species occurring naturally is the common alder (*Alnus glutinosa* (L.) Gaertn.). This species is widespread in the northern and central parts of the country, mainly in the flooded plains and swamps at lower altitudes than the mountain riparian systems often associated with other broadleaved tree species such as *Quercus* spp. and *Salix* spp. [3].

Since the early 1990s, alder ecosystems have been severally impacted by an emerging disease that has contributed to their decline and regression in the European continent and some areas of North America [4,5]. Typical symptoms include general or progressive canopy dieback, stem bleeding cankers, necrotic bark lesions at the collar and root rot [6].

Many studies have investigated the causes of this disease, identifying the causal agents as some members of the *Phytophthora* genus [4,7,8]. The disease appears to have an extremely complex aetiology; independent surveys have ascertained the occurrence of over 30 species of *Phytophthora* in declining alder ecosystems between North America and Europe [5,7,9,10]. Many of these species belong to the *Phytophthora* clade 6 *sensu* [11]. This clade includes organisms closely related to aquatic environments. The ecology of several *taxa* is still unclear; some species are known to have a saprotrophic or opportunistic lifestyle, while a few are reported to be aggressive pathogens [12,13].

Among the other *Phytophthora* species associated with declining alder trees, several belong to clade 2. *Phytophthora plurivora* is one of the most widespread species in declining alder ecosystems in Europe, and its pathogenicity has been confirmed using different inoculation techniques [8,14,15]. In contrast, in North America, *P. siskiyouensis* is reported as one of the most aggressive pathogens of the *Alnus* species [16,17].

Despite the studies that have been conducted in Europe during the last three decades, many issues about the aetiology of alder decline remain to be clarified, as well as the distribution and impact of the different *Phytophthora* species among the countries. In Portugal, until now, only one study has investigated the role of *Phytophthora* species in alder decline [18]. The study was conducted on two alder stands along two rivers in central Portugal, confirming the involvement of two species, *Phytophthora* × *alni* and *P. lacustris*, in the disease aetiology.

Therefore, given the still limited information on the occurrence and impact of *Phytophthora* species in Portuguese riparian habitats and the recent discovery in central Portugal of five riparian ecosystems with high mortality rates of common alder trees, a study was conducted to establish the causal agents and obtain new data about the diversity and impact of *Phytophthora* species.

## 2. Materials and Methods

### 2.1. Field Surveys and Sampling Procedure

Monitoring activities were conducted during spring 2022 on five natural *Alnus glutinosa* stands located in the central part of Portugal, the districts of Aveiro and Guarda (Table 1). The altitude of survey sites ranged from 9 to 750 m. a.s.l.

At each site, mature alder trees were visually checked for the presence of typical *Phytophthora* disease symptoms, including wilting of foliage, shoot and twigs dieback, sudden death, bleeding cankers, and root and collar rot. In Sites 2 and 3, four linear transects of 50 m were randomly established to evaluate disease incidence and mortality rate, expressed as the number of symptomatic trees out of the total number of trees (DI = n/N × 100) and the number of dead trees out of the total number of trees (M = d/N × 100), respectively [19].

At each site, representative trees were randomly chosen for sampling (Table 1). Rhizosphere soil samples (about 1 L of soil and fine roots) were collected around the collar of 38 declining alder trees. Among these, eight trees were chosen for the collection of bark tissue samples, taking small fragments from the border of bleeding cankers on the stem. In Sites 2 and 3, the occurrence of *Phytophthora* species was also monitored in the water streams using nylon mesh bags containing 10 young cork oak (*Quercus suber* L.) leaves as bait [10,20]. The nylon mesh bags were positioned near the root systems of the selected alder trees.

### 2.2. Isolation and Identification of Phytophthora Species

In the laboratory, samples were processed to isolate the pathogens in pure culture. Rhizosphere samples were placed in plastic boxes and flooded with 2 L of distilled water. After 24 h, pittosporum (*Pittosporum* sp.) leaves were placed on the water surface and used as bait. Boxes were kept at 18–20 °C under natural daylight, and after 3–5 days, leaves showing dark spots were cut into small pieces (5 mm^2^) and placed on Petri dishes containing the selective medium PDA+ [21].

Isolation of *Phytophthora* species was also performed directly from the necrotic tissues, taking small inner bark fragments along the border of the bleeding cankers with a sterile scalpel in aseptic conditions and placing them in Petri dishes containing PDA+.

After ten days, the mesh bags, floating on the water surface, were collected from the stream and transferred to the laboratory. Leaves showing necrotic dark spots were cleaned in sterile distilled water for 10 s, dried on sterile papers, cut into small fragments, and used for isolation of *Phytophthora*, as illustrated above.

The isolates in pure culture were initially grouped in morphotypes and identified based on the colony appearance after 7 days on potato dextrose agar (PDA) and carrot agar (CA) at 20 °C in the dark, the presence/absence of chlamydospores and hyphal swelling, the biometric data of sporangia produced on CA plugs floating in unsterile water in the Petri dishes and breeding systems, as reported by Bregant et al. [10]. All isolates were preserved in glycerol at −80 °C at the Department of Biology, University of Aveiro, Portugal; some representative isolates of each species are stored on PDA and CA slants under oil in the culture collection of the Dipartimento Territorio e Sistemi Agro-Forestali, Università degli Studi di Padova, Italy.

### 2.3. Identification of Isolates

The identity of all isolates was confirmed by analyses of the DNA sequences. The genomic DNA of the isolates was extracted from the mycelium of 5-day-old cultures grown on PDA at 20 °C, according to the protocol reported by Möller [22]. The rDNA internal transcribed spacer region (ITS) was the locus chosen to be sequenced to identify the isolates. The primers ITS5 and ITS4 were used to amplify and sequence the entire ITS region, including the complete 5.8S gene [23]. Polymerase chain reactions (PCRs) were performed in a final volume of 25 mL reaction mixtures containing 15.75 μL of molecularly pure water, 6.25 μL of NZYTaq 2× green Master Mix (NzytechTM, Lisbon, Portugal), 1 μL of each primer at 10 pmol/μL and 1 μL of the DNA template. PCR amplification conditions were performed as described by Linaldeddu et al. [24] in a Bio-Rad C1000 touch thermal cycler (Bio-Rad Laboratories, Inc., Hercules, CA, USA). The nucleotide sequences were read and edited with FinchTV 1.4.0 (Geospiza Inc., Seattle, WA, USA). and then compared with reference sequences (ex-type culture or representative strains) retrieved in GenBank using the BLAST search function [25]. Isolates were assigned to a species when their sequences were identical (100%) to the sequence of type material or representative isolates (Table 2). Sequences from representative isolates of each species were deposited at GenBank (Table 2).

### 2.4. Pathogenicity Test

To confirm Koch’s postulates for new host–pathogen associations, the pathogenicity of five *Phytophthora* species was tested by inoculation on 1-year-old common alder seedlings grown in plastic pots (5 cm diameter, 0.5 L volume). Ten seedlings were inoculated with a representative isolate of each species, and ten were used as control. The seedlings were inoculated by wounding at the base of the stem using the protocol reported by Bregant et al. [10].

All inoculated seedlings were kept in controlled conditions at 21 °C and watered regularly for 30 days. At the end of the experimental period, seedlings were checked for the presence of internal (necrotic lesion) and external (wilting and exudates) disease symptoms. For each seedling, the outer bark was carefully removed with a scalpel, and the length of the necrotic lesion surrounding each inoculation point was measured.

The re-isolation of isolates was attempted by transferring 5 pieces of inner bark taken around the margin of the necrotic lesions onto PDA+. Growing colonies were subcultured onto CA and PDA, incubated in the dark at 20 °C and identified by morphological and molecular analyses.

### 2.5. Data Analysis

Pathogenicity assay data were checked for normality and then subjected to analysis of variance (ANOVA). Significant differences among mean values were determined using Fisher’s least significant differences multiple range test (*p* = 0.05) after one-way ANOVA using XLSTAT 2008 software (Addinsoft, Paris, France).

## 3. Results

### 3.1. Symptomatology

Field surveys conducted in five common alder ecosystems in Portugal showed the widespread occurrence of severe Phytophthora disease symptoms on young and old alder trees. Severe disease symptoms were observed mainly in the periodically flooded areas. Disease incidence, calculated at Sites 2 and 3, ranged from 75% to 83%, with an average mortality rate of 32%.

**Figure 1:**
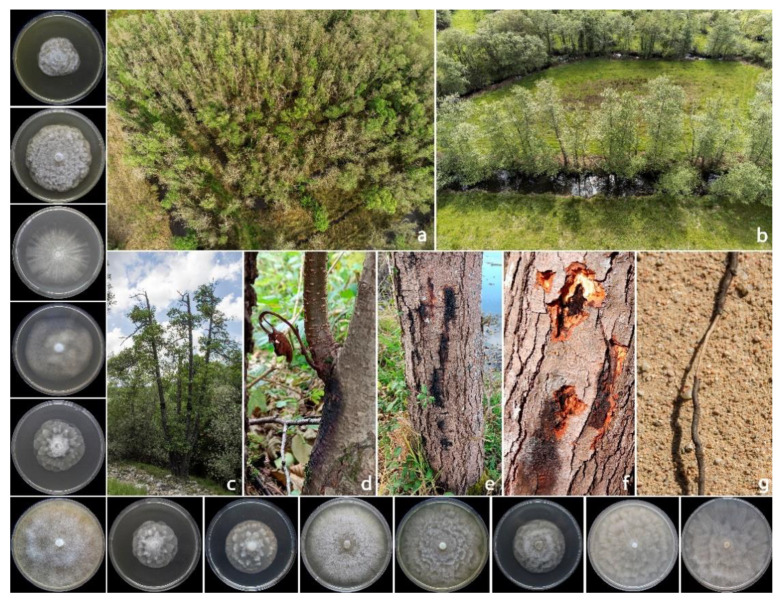
Overview of *Phytophthora*-related diseases on *Alnus glutinosa*: panoramic view that highlights a high mortality rate (**a**); alders with initial declining symptoms along a stream (**b**); severe branch dieback symptoms (**c**); bleeding cankers on stems with a wilted shoot (**d**); bleeding cankers (**e**,**f**) and root rot (**g**). On the left, starting from the top, colony morphology of: *Phytophthora amnicola*, *P. asparagi*, *P. bilorbang, P. cactorum*, *P. chlamydospora, P. cinnamomi, P. gonapodyides, P. lacustris, P. multivora, P. plurivora, P. polonica, P. pseudocryptogea* and *P. rosacearum* after 7 days of growth at 20 °C on CA in the dark.

Declining trees were characterized by complex symptomatology, including extensive bleeding cankers on the lower part of the stem and, sometimes, on the branches, with irregular-shaped, inner bark reddish-brown necrosis as the result of the death of the bark tissues (Figure 1). In addition, different canopy symptoms, such as rusty shrivelled leaves, small-size leaves, shoot blight and epicormic shoots, were observed. In the late stage of the disease, infection causes a progressive or sudden decline of the whole canopy.

### 3.2. Aetiology

A total of sixty-three *Phytophthora* isolates were obtained from 60 out of 66 processed samples (positivity 90.1%). Among these, 4 isolates were obtained from bleeding cankers, 36 from rhizosphere (fine roots) and 23 from leaves used as bait along the streams. Based on morphology, colony appearance and DNA sequence data for 13 *Phytophthora* species, namely, *P. lacustris* (13 isolates), *P. multivora* (10), *P. amnicola* (9), *P. chlamydospora* (6), *P. polonica* (6), *P. bilorbang* (4), *P. plurivora* (4), *P. cinnamomi* (3), *P. asparagi* (2), *P. cactorum* (2), *P. pseudocryptogea* (2), *P. gonapodyides* (1) and *P. rosacearum* (1), were identified (Table 2).

The most common *Phytophthora* species isolated in this study was *P. lacustris*; this species was obtained mainly from water streams. *Phytophthora amnicola* and *P. multivora* were the dominant species in rhizosphere samples, whereas *P. multivora* was the only species occurring in all types of samples (bark tissue, rhizosphere and water). *Phytophthora chlamydospora* and *P. plurivora* were the most widespread species, occurring in three sites (Table 2). Seven out of thirteen species isolated belonged to *Phytophthora* ITS clade 6, the most represented clade, followed by clade 2.

### 3.3. Pathogenicity

**Figure 2:**
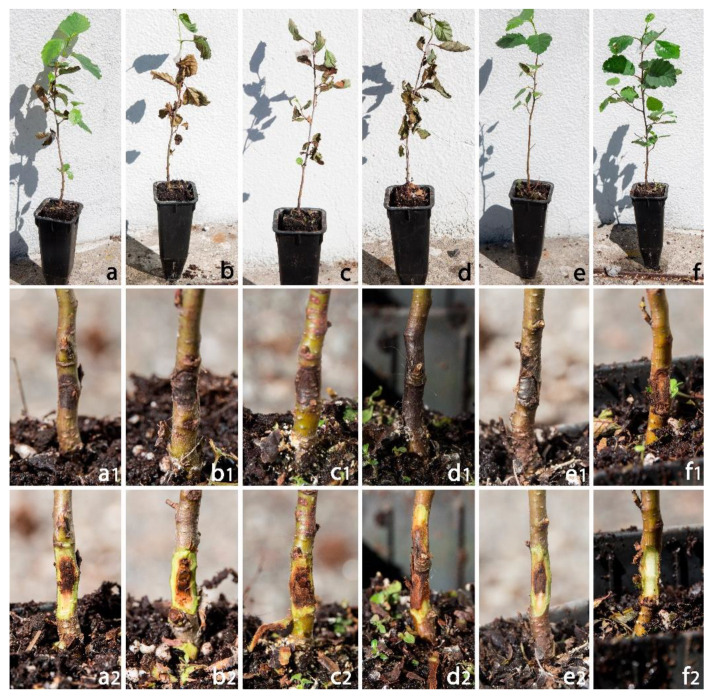
Symptoms observed on common alder seedlings after 30 days from inoculation with *Phytophthora amnicola* (**a**–**a2**), *P. asparagi* (**b**–**b2**), *P. chlamydospora* (**c**–**c2**), *P. multivora* (**d**–**d2**) and *P. rosacearum* (**e**–**e2**). Control seedlings (**f**–**f2**).

At the end of the experimental period, *Phytophthora*-inoculated alder seedlings showed severe wilted symptoms associated with dark brown inner bark lesions that spread up and down from the inoculation point (Figure 2). All inoculated *Phytophthora* species proved to be pathogenic on common alder.

The average lesion length differed significantly among species (Table 3). The lesions caused by *P. multivora* were significantly larger than those caused by the other species. Necrotic inner bark lesions caused by *P. multivora*, *P. chlamydospora*, *P. asparagi* and *P. amnicola* progressively girdled the stem, causing wilting symptoms and the sudden death of the seedlings (Table 3). Control plants inoculated with sterile PDA plugs remained symptomless. All inoculated species were successfully re-isolated (100%) from the margin of the necrotic inner bark lesions of all seedlings. No *Phytophthora* isolates or other microorganisms were re-isolated from control seedlings.

## 4. Discussion

An extensive survey of *Phytophthora*-related diseases, conducted in central Portugal, showed the occurrence of 13 species, *P. amnicola*, *P. asparagi*, *P. bilorbang*, *P. cactorum*, *P. chlamydospora*, *P. cinnamomi*, *P. gonapodyides*, *P. lacustris*, *P. multivora*, *P. plurivora*, *P. polonica*, *P. pseudocryptogea* and *P. rosacearum,* in five riparian habitats (rivers and streams) characterized by high common alder mortality. Ten out of thirteen species were recovered from naturally declining common alder ecosystems in previous studies in Europe [7,8,9,10,26,27].

Most species found in this study are classified in the ITS clade 6 *sensu* [11,28]. This major clade includes a large number of saprotrophic or weak opportunistic pathogens strongly linked to aquatic environments [12]; therefore, it is not surprising that *P. lacustris* was found to be the dominant species. Its presence in Portugal, associated with declining alder formations, was recently documented by Kanoun-Boulè [18]. Three of the other species belonging to clade 6, *P. bilorbang*, *P. chlamydospora* and *P. gonapodyides,* are very common in the temperate riparian habitats of Europe and other continents, often in association with declining alders [9,10,29] whereas *P. amnicola, P. asparagi* and *P. rosacearum* are reported here for the first time on declining common alder.

*Phytophthora amnicola* was originally described in 2012 in Western Australia [30]. Its lifestyle appears strongly related to water, this is corroborated by the data obtained in this survey, but little is known regarding the ecology and potential impact of this species in Portugal.

From the rhizosphere of two alder trees, the colonies of *P. asparagi* were obtained. This species was described in the USA in 2012, but it has been known for a long time and the name is currently considered invalid [31,32]. *Phytophthora asparagi* has been reported in different countries as a pathogen on ornamental and crop plants [31,33]. Some recent studies have demonstrated that this species is widespread in Mediterranean environments on several species of the Mediterranean maquis [34,35]. Its diffusion in natural areas appears closely linked to the white asparagus (*Asparagus albus*), a preferential host that can facilitate host jumps [34].

*Phytophthora rosacearum* was isolated for the first time in California on *Malus* sp. and later from other diseased crops, such as pear in California and pomegranate in Turkey [36,37,38]. The recovery of *P. rosacearum* on common alder in Portugal represents the first report of this species in a natural habitat and in Europe. Based on the results obtained in the pathogenicity test, *P. rosacearum* can be considered a weak pathogen of common alder compared to other *Phytophthora* species.

The second most represented clade consists of two species, *P. multivora* and *P. plurivora*. *Phytophthora multivora* was the only species found in all types of samples monitored (stem bleeding cankers, necrotic fine roots and baited leaves along the stream). The discovery of this polyphagous pathogen causing the root rot and mortality of common alder in Portugal poses an additional threat to alder stands in Europe. *Phytophthora multivora* was previously reported to cause root and collar rot lesions on *Agathis australis*, *Agonis flexuosa*, *Banksia* spp., *Corymbia calophylla*, *Eucalyptus* spp., *Rubus anglocandicans* and *Wollemia nobilis* in Australia and New Zealand and on *Acacia mearnsii*, *Alnus glutinosa*, *Araucaria araucana*, *Quercus* spp., *Rhododendron* sp. and *Salix fragilis* in natural areas in Chile, Czech Republic, Germany, Hungary, New Zealand and South Africa [39,40,41,42,43,44,45,46,47,48]. The underbark inoculation test confirmed the aggressiveness of this emerging pathogen on common alder. Previous studies have ascertained its pathogenicity on *Agathis australis*, *Agonis flexuosa*, *Corymbia* spp., *Eucalyptus* spp., *Banksia* spp., *Rubus anglocandicans* and *Wollemia nobilis* [40,42,44,45,49,50].

The other species consistently detected from bark lesions and rhizospheres was *P. plurivora*, a plurivorous pathogen involved in the aetiology of several diseases of woody hosts in six continents [51,52,53,54]. The high isolation frequency of *P. plurivora* in this study is in accordance with the results of previous studies conducted on declining common alder trees in Italy [8,10].

Finally, the other four species were recovered less frequently. Among these, *P. cactorum, P. polonica* and *P. pseudocryptogea* are detected for the first time and are related to declining forests in Portugal; however, they are already known in common alder in other European countries [10,55]. The discovery of *P. cinnamomi* in riparian habitats is of particular concern due to its wide host range and the impact of this pathogen on oak forests in Portugal [56].

The fulfilment of Koch’s postulates for the five species tested in this study expands the list of pathogenic *Phytophthora* species for common alder to 29 (Table S1), suggesting that the disease may be caused by more than one pathogen under different environmental conditions.

## 5. Conclusions

While *P. × alni* has been getting the most attention in the last decades, the most common species isolated from declining alder trees in Europe is *P. plurivora* [9,10,27]. At the same time, the current trend of discovering an increasing number of pathogenic species in declining alder trees emphasizes how much more we need to learn about *Phytophthora* biodiversity and their impact on riparian ecosystems. In general, this work contributes to expanding knowledge on the biodiversity of *Phytophthora* species in the natural areas of Portugal with eight new reports (Table S2).

## Figures and Tables

**Table 1:** Study sites’ information and number of stem (S), rhizosphere (R) and leaf (L) samples used for *Phytophthora* isolation.

Survey Sites	Elevation (m a.s.l.)	Geographic Coordinates		Number of Samples
1	9	40.7035888	−8.6052296	R(4)
2	11	40.7206700	−8.5652620	R(20), S(5), L(10)
3	11	40.7141470	−8.5738595	R(10), S(3), L(10)
4	417	40.6128810	−7.5174910	R(2)
5	750	40.4106660	−7.4713180	R(2)

**Table 2:** *Phytophthora* isolates obtained from stem (**S**), rhizosphere (**R**) and water (**W**) samples in the investigated sites.

Species	Accession Number	ITS Clade	Number of Samples			Sites
			Stem	Rhizosphere	Water	
*P. amnicola*	OQ202216	6	-	6	3	2,3
*P. asparagi*	OQ202217	6	-	2	-	3
*P. bilorbang*	OQ202218	6	-	-	4	2,3
*P. cactorum*	OQ202219	1	-	2	-	2
*P. chlamydospora*	OQ202220	6	-	4	2	2,3,4
*P. cinnamomi*	OQ202221	7	-	3	-	2
*P. gonapodyides*	OQ202222	6	-	1	-	3
*P. lacustris*	OQ202223	6	-	4	9	2,3
*P. multivora*	OQ202224	2	3	6	1	2,3
*P. plurivora*	OQ202225	2	1	3	-	1,2,4
*P. polonica*	OQ202226	9	-	2	4	2,3
*P. pseudocryptogea*	OQ202227	8	-	2	-	3,5
*P. rosacearum*	OQ202228	6	-	1	-	2

**Table 3:** Mean lesion length ± standard deviation caused by each *Phytophthora* species on the stem of common alder seedlings and the percentage of seedlings with exudates and wilting symptoms.

Species	Isolates	Mean Lesion Length (mm) *	Exudates	Wilting	Re-Isolation (%)
*P. amnicola*	CBP28	11.0 ± 4.8bc	-	30%	100
*P. asparagi*	CBP23	12.2 ± 4.7bc	-	40%	100
*P. chlamydospora*	CBP16	15.5 ± 4.8b	-	30%	100
*P. multivora*	CBP56	41.2 ± 14.7a	40%	80%	100
*P. rosacearum*	CBP81	8.5 ± 3.1cd	-	-	100
Control	-	2.5 ± 1.4d	-	-	-
Critical value	-	2.006			

* Values in the column with the same letter do not differ significantly at *p* = 0.05, according to the LSD multiple range test.

## Supplementary Materials

The following supporting information can be downloaded at: https://www.mdpi.com/article/10.3390/pathogens12020276/s1, Table S1: *Phytophthora* species reported in natural ecosystems in Portugal; Table S2: *Phytophthora* species reported as pathogenic on *Alnus glutinosa* [57,58,59,60,61,62,63,64,65,66,67,68,69,70,71,72,73,74].
