# Supplementary material for: Phytophthora Species Involved in Alnus glutinosa Decline in Portugal
Source: Pathogens. 2023 Feb 8;12(2):276. doi: 10.3390/pathogens12020276 (PMC9966130; doi:10.3390/pathogens12020276)
Supplement: Supplementary file 1 [file pathogens-12-00276-s001.zip › pathogens-2194170-supplementary.pdf]

pathogens

Table S1. *Phytophthora* species reported in natural ecosystems in Portugal.

Species	Host	References
P. alticola	*Eucalyptus globulus*	[57]
P. amnicola	*Alnus glutinosa*	This study
P. asparagi	*A. glutinosa*	This study
P. bilorbang	water	This study
P. cactorum	*A. glutinosa*	This study
P. cambivora	*Castanea sativa*	[58,59]
P. castanetorum	*C. sativa*	[60]
P. chlamydospora	*A. glutinosa*	This study
P. cinnamomi	*A. glutinosa, Arbutus unedo, Calluna vulgaris, Castanea sativa, Cistus crispus, C. ladanifer, C. populifolius, C. salvifolius, E. globulus, Genista triacanthos, Pinus pinaster, Quercus rotundifolia, Q. robur, Q. suber, Ulex* spp.	[56,57,61–64]; this study
P. condilina	water	[65]
P. gonapodyides	*A. glutinosa*, water	[65]; this study
P. inundata	water	[65]
P. lacustris	*A. glutinosa*	[18]
P. multivora	*A. glutinosa*	This study
P. plurivora	*A. glutinosa*, water	[65], this study
P. polonica	*A. glutinosa*	This study
P. pseudocryptogea	*A. glutinosa*, water	[65], this study
P. psychrophila	*Q. rotundifolia*	[66]
P. quercina	*Q. rotundifolia, Q. pyrenaica*	[60]
P. rosacearum	*A. glutinosa*	This study
P. ramorum	*Viburnum* sp.	[67]
P. ×alni	*A. glutinosa*	[18]

Table S2. *Phytophthora* species reported as pathogenic on *Alnus glutinosa*.

**Species**	**References**
P. amnicola	This study
P. asparagi	This study
P. acerina	[8]
P. cactorum	[7]
P. cambivora	[68,69]
P. chlamydospora	This study
P. cinnamomi	[68]
P. citrophthora	[70]
P. cryptogea	[68]
P. gallica	[71]
P. gonapodyides	[7]
P. inundata	[72]
P. lacustris	[7,72]
P. megasperma	[7]
P. multivora	This study
P. nicotianae	[70]
P. palmivora	[70]
P. plurivora	[7,8,14,15,73]
P. polonica	[55]
P. pseudocryptogea	[8]
P. pseudosyringae	[74]
P. ramorum	[73]
P. rubi	[68]
P. syringae	[55]
P. rosacearum	This study
P. uniformis	[15,68]
P. ×alni	[15,68]
P. ×multiformis	[15,68]
Phytophthora taxon raspberry	[72]
